# Herpesvirus Exploitation of Host Immune Inhibitory Pathways

**DOI:** 10.3390/v4081182

**Published:** 2012-08-03

**Authors:** Gabrielle Stack, Maria A. Stacey, Ian R. Humphreys

**Affiliations:** Institute of Infection and Immunity, Cardiff University, Cardiff, CF14 4XN, UK; Email: stackg@cf.ac.uk (G.S.); staceyma1@cf.ac.uk (M.A.S.)

**Keywords:** herpesvirus, immune suppression, IL-10, CD200, PD-1, BTLA, cytomegalovirus

## Abstract

Herpesviruses employ a plethora of mechanisms to circumvent clearance by host immune responses. A key feature of mammalian immune systems is the employment of regulatory pathways that limit immune responsiveness. The primary functions of these mechanisms are to control autoimmunity and limit exuberant responses to harmless antigen in mucosal surfaces. However, such pathways can be exploited by viral pathogens to enable acute infection, persistence and dissemination. Herein, we outline the current understanding of inhibitory pathways in modulating antiviral immunity during herpesvirus infections *in vivo* and discuss strategies employed by herpesviruses to exploit these pathways to limit host antiviral immunity.

## 1. Introduction

The mammalian immune system has evolved to encompass activating and inhibitory pathways. These counter-regulatory mechanisms enable the immune system to initiate protective coordinated responses against invading pathogens that are sufficiently limited in magnitude to avoid overt bystander damage to infected tissues. In addition, inhibitory immune pathways limit the development of harmful autoimmune reactions and over-exuberant immune responses to harmless antigens that are often encountered at mucosal surfaces. 

Herpesviruses are large DNA viruses that are divided into the α-herpesviridae, β-herpesviridae and γ-herpesviridae families. Herpesviruses establish life-long infection in their hosts and are ubiquitous within the human population. These infections are often asymptomatic in healthy individuals. However, herpesvirus infections cause significant disease in immune compromised individuals, and the β-herpesvirus human cytomegalovirus (HCMV) is the leading infectious cause of congenital defects in the Western world. Moreover, the γ-herpesvirus Epstein Barr virus (EBV) causes infectious mononucleosis and, in addition to the γ-herpesvirus Kaposi's sarcoma-associated herpesvirus (KSHV), is associated with the development of several cancers [1]. 

Herpesviruses are thought to have emerged approximately 400 million years ago [2]. During co‑evolution with mammals, herpesviruses have acquired numerous genes encoding proteins that function primarily to evade or manipulate immune pathways, thus enabling the establishment of life‑long infection within their host. It is becoming increasingly apparent that host immune inhibitory pathways critically influence the outcome of herpesvirus infections. In this article, we summarize current understanding of the immune inhibitory mechanisms that modulate anti-herpesvirus immunity and highlight strategies adopted by herpesviruses to exploit such pathways to antagonize antiviral immunity. 

## 2. Mammalian and Viral Interleukin-10

The immune-modulatory cytokine interleukin-10 (IL-10) is a member of the same family of proteins as type I IFNs and IFNγ [3]. Mammalian IL-10 is produced predominantly by macrophages, dendritic cells (DCs), B cells and regulatory T cells (Tregs), although it can be produced by virtually all T cell subsets [4]. The IL-10 receptor (IL-10R) is composed of two subunits. The IL-10R1 (IL-10Rα) is the ligand-binding subunit expressed by hematopoietic cells whereas IL-10R2 (IL‑10Rβ) is an accessory subunit for signaling constitutively expressed by most hematopoietic and non-hematopoietic cells. The interaction between IL-10 and its receptor engages the Jak family tyrosine kinases Jak1 and Tyk2, which are constitutively associated with IL-10R1 and IL-10R2 respectively, leading to tyrosine phosphorylation and activation of STAT (signal transducers and activators of transcription) proteins, predominantly STAT3 and to a lesser extent STAT1 and STAT5. 

IL-10 can induce a broad range of biological functions (reviewed in [5,6]). Although IL-10 displays certain immune-stimulatory activities, the majority of data to date demonstrates that IL-10 exerts suppressive effects on immune cells, particularly APCs and T cells (either directly or via suppression of APC function). Consequently, IL-10 profoundly influences a plethora of immune responses *in* *vivo*, including responses elicited in response to parasitic and bacterial infections [7]. 

During acute viral infections, IL-10 has paradoxical functions. For example, IL-10 can limit immunopathology induced by respiratory syncytial virus (RSV) [8,9], and influenza [10], suggesting the primary function of this pathway is to limit infection-induced immune-mediated tissue damage. Following high dose influenza infection however, IL-10-deficient mice exhibit accelerated clearance of virus which is associated with the induction of antiviral Th17 cells [11], suggesting that the biological outcome of IL-10R signaling during acute infection may vary depending on the virus pathogen and the infectious dose. Importantly, during chronic virus infection *in vivo*, as demonstrated in the LCMV model, IL-10 antagonizes antiviral immunity and promotes virus persistence [12,13]. 

### 2.1. EBV BCRF1

The importance of IL-10R inhibition of anti-herpesvirus immunity is implied by the evolutionary acquisition of functional IL-10 homologues (vIL-10) by herpesviruses [14,15,16,17,18], including the human viral pathogens HCMV [19] and EBV [20]. The EBV-encoded protein BCRF1 was the first herpesvirus-encoded protein identified to contain sequence homology (90% at amino acid level) with mammalian (human) IL-10 [20]. BCRF1 is expressed during lytic infection [21,22]. It inhibits monocyte stimulation of T cells [23], and antagonizes expression of MHC class II [23] and costimulatory ligands [24] on the surface of macrophages/monocytes, suggesting BCRF1 functions to inhibit the activation of virus-specific CD4^+^ T cells. Further, BCRF1 treatment of B cells inhibits expression of the transporter protein TAP1 and the immunoproteosome subunit b1i/LMP2 [25], interfering with B cell translocation of antigenic peptides to the endoplasmic reticulum for loading onto MHC class I. Thus, during active replication in B cells BCRF1 may contribute to evasion of direct recognition by CD8^+^ T cells. BCRF1 may also exert more broad suppressive effects on the antiviral immune response via inhibition of cytokine production by T cells [20,26] and monocytes [23].

Like mammalian IL-10, BCRF1 also exhibits immune stimulatory properties *in vitro*. Specifically, BCRF1 promotes B cell immunoglobulin secretion [27], survival [28] and proliferation [29] in a comparable manner to mammalian IL-10. Interestingly however, BCRF1 lacks certain properties of mammalian IL-10 including the ability to induce mast cell proliferation and expression of MHC class II by B cells [29]. The presence of an isoleucine residue at position 87 of hIL-10 is critical for immunostimulatory functions of IL-10. Interestingly, this residue is replaced by an alanine in BCRF1 and an alanine > isoleucine substitution in this position partially reconstitutes the BCRF1 stimulatory functions exhibited by mammalian IL-10 [30]. Importantly, BCRF1 demonstrates a 1,000-fold reduction in affinity for IL-10Rα [31]. Although a CD4^+^ T cell clone displays reduced sensitivity for BCRF1‑mediated inhibition of IL-2 production as compared to mammalian IL-10 [31], the reduced affinity has little influence on other suppressive activities of BCRF1 [20,23,24,25,26]. Thus, BCRF1 may selectively exploit the immune suppressive functions of the IL-10R pathway for the purpose of evading host immunity and establishing persistent/latent infection. Further, the ability to induce B cell transformation [32] and B cell expansion/survival [28,29] implies that BCRF1 may promote carriage of latent virus in B cells. 

### 2.2. HCMV UL111A

The HCMV *UL111A* gene product shares 27% amino acid sequence identity with mammalian IL-10 [19]. Despite this relatively low homology, the UL111A-encoded protein binds hIL-10Rα with higher affinity than mammalian IL-10 [33]. *In vitro* studies demonstrated that UL111A (or cmvIL-10), which is expressed during lytic infection [19], displays many of the immunomodulatory functions of mammalian IL-10 (reviewed in [34]) including inhibition of macrophage/monocyte activation and pro-inflammatory cytokine production [35] and dendritic cell maturation and survival [36,37]. Thus, these experiments suggest that UL111A dampens virus-induced innate immune cell activation and subsequent priming of adaptive immunity.

Intriguingly, UL111A expression is also detected in natural and experimental latent infection [38]. Latency-associated UL111A transcript (termed LAcmvIL-10) is differentially spliced, containing only the first two introns of UL111A. Subsequently, LAcmvIL-10 lacks certain functional characteristics of full-length UL111A including the induction of STAT3 phosphorylation, suppression of DC maturation [38] and enhancement of Fc-mediated phagocytosis by monocytes [39]. Paradoxically, LAcmvIL-10 maintains the ability to suppress monocyte activation [38] suggesting that HCMV may express LAcmvIL-10 during latent infection to subvert host immunity during this phase of infection. In support of this hypothesis, myeloid progenitor cells infected with AD169 strain HCMV lacking the UL111A gene (and subsequently the full-length and truncated transcripts) exhibit heightened MHC class II expression, as compared to cells infected with wild type virus, and elicit a superior alloreactive T cell response [40]. Thus, UL111A gene products may dampen host immune responsiveness during acute *and* latent CMV infection *in vivo*. 

### 2.3. Mammalian IL-10 and Herpesvirus Infections

As discussed, mammalian IL-10 is an important regulator of immune responses elicited by a plethora of virus infections. The role that mammalian IL-10 plays in regulating anti-herpesvirus immunity *in vivo* has been elucidated in murine systems, particularly the murine cytomegalovirus (MCMV) model of infection. IL-10 is expressed by numerous hematopoietic cells during acute MCMV infection [41,42]. IL-10 deficient mice suffer from more severe MCMV-induced disease than wild type mice [43], including increased weight loss driven by an over-exuberant TNF-producing T cell response [44]. Interestingly, IL-10 inhibition of natural killer (NK) cell:DC crosstalk is critically required to control this pathological T cell response [44]. Furthermore, intracranial MCMV infection causes lethal disease in the absence of IL-10; a phenotype associated with heightened pro‑inflammatory cytokine production rather than ineffective control of virus replication [45]. In the situation of uncontrolled virus replication in mice lacking perforin, IL-10 is secreted by (NK) cells and suppresses pathogenic CD8^+^ T cell responses [41]. In this context, IL-10 neutralization increases virus load suggesting that IL-10 can limit MCMV replication, although the mechanism(s) by which this occurs is unclear [41]. Interestingly, during acute limiting MCMV infection IL-10 *promotes* the accumulation of NK cells by limiting NK cell activation and subsequent activation-induced cell death [42]. Taken with data in a murine model of ocular herpesvirus simplex infection where murine IL‑10 dampens infection-induced myeloid and lymphoid inflammation [46], these data are consistent with the idea that, via its suppressive properties, mammalian IL-10 functions to protect the host during acute herpesvirus infection by limiting over-exuberant and self-destructing immune responses elicited in response to virus challenge.

IL-10R signaling during chronic herpesvirus infection antagonizes protective antiviral immunity. Persistent MCMV replication in the salivary glands is associated with the presence of localized CD4^+^ T cells [47] and NK cells [48] capable of expressing IL-10. Therapeutic blockade of IL-10R signaling during this persistent phase of infection enhances Th1 cell accumulation within the salivary glands and accelerates virus clearance [47]. In addition, IL-10 deficient mice mount heightened CD4^+^ and CD8^+^ T cell responses during the chronic/latent stage infection and harbor reduced levels of viral DNA load in infected mucosal (lungs) and non-mucosal (spleen) organs as compared with wild type mice [49]. Therapeutic blockade of IL-10R during reactivation of latent γ-herpesvirus-68 infection in immune compromised (CD4-depleted) mice also leads to a reduction in virus load [50]. Therefore, although IL-10 inhibition of T cell responses primed during acute infection can lead to an increase of virus load during MCMV persistence [44], studies to date collectively suggest that mammalian IL-10 can act during both the chronic replicating and latent stages of herpesvirus infections to antagonize antiviral immunity, thus contributing to carriage of herpesviruses within their mammalian hosts.

Given the critical role of mammalian IL-10 in the suppression of anti-herpesvirus immunity, why have certain herpesviruses acquired and retained IL-10 homologues? Clearly the kinetics and tropism of expression of mammalian and viral IL-10 molecules may differ, and structural and functional deviations of vIL-10s from their mammalian counterparts may have profound effects *in vivo*. Thus, vIL-10 molecules may exert complimentary and/or differential effects to mammalian IL-10 that are evolutionarily advantageous for the virus. Peter Barry and colleagues have shed substantial light on this subject in a series of elegant studies using rhesus CMV (rhCMV) which, unlike other experimental models of CMV infection, encodes a functional IL-10 homologue (UL111A). Subcutaneous infection with a ΔUL111A virus elicited a greater macrophage infiltrate into the site of infection than that observed in response to wild type virus, and ΔUL111A-infected macaques displayed heightened dendritic cell accumulation in draining lymph nodes. ΔUL111A rhCMV also elicited increased virus‑specific T cell responses and a larger, more rapid humoral immune response than wild type virus [51]. These experiments clearly demonstrate, as predicted by the large number of *in vitro* and *in vivo* studies using different models of CMV infection, a clear benefit for cytomegalovirus to specifically target the IL-10R signaling pathway to dampen antiviral immunity. Although the authors reported no obvious effect of UL111A deletion on virus secretion in saliva and urine during acute experimental infection [51], the retention of this vIL-10 by rhCMV (and other herpesviruses) suggests that these cytokine homologues offer substantial benefit(s) for the virus in promoting their survival and/or dissemination.

## 3. Programmed Death Receptor (PD-1)

The Programmed Death Receptor (PD-1) is a member of the CD28 family expressed on CD4^+^, CD8^+^ and NK T cells, B cells, monocytes and on some dendritic cell subsets upon activation. It is a type 1 transmembrane glycoprotein with an IgV-type extracellular domain and a cytoplasmic signaling domain containing two tyrosine residues [52]. It is a monomeric receptor which has two ligands, PD-L1 (B7-H1, CD274) [53] and PD-L2 (B7-DC, CD273) [54]. PD-L1 expression is detectable on resting B, T, myeloid and dendritic cells and can be up-regulated upon activation [55] as well as on non-lymphoid tissues such as heart, skeletal muscle, placenta and lung tissues [53]. PD-L2 has significant homology to PD-L1 (38% amino acid identity in mice [56]) and is expressed in an inducible manner on dendritic cells, macrophages [54] and activated T cells [57]. 

**Figure 1:**
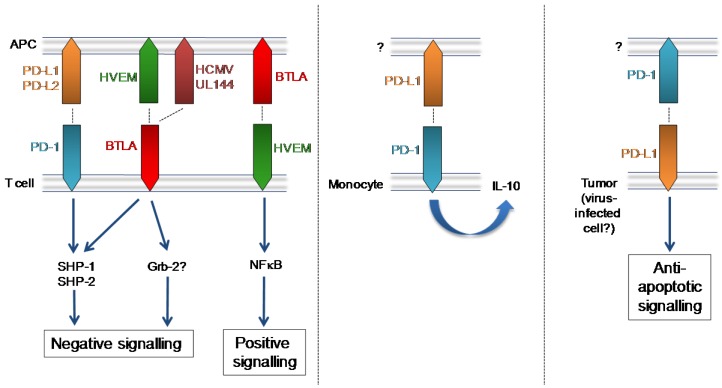
PD-1, BTLA and their mammalian and herpesvirus ligands. Left: T cell-expressed PD-1 and BTLA induce inhibitory signalling following binding of their respective mammalian and viral ligands. BTLA binding to T cell-expressed HVEM elicits NFκB‑induced pro-inflammatory/pro-survival signalling. Middle: Up-regulation of PD-1 expression by monocytes is observed in HIV infection and PD-1 ligation induces monocyte secretion of IL-10. Right: PD-L1 expressed by tumour cells induces anti-apoptotic signalling.

The binding of PD-1 to its ligands induces an inhibitory signal within the PD-1-expressing cell [56,58] thought to be triggered by immunoreceptor tyrosine-based inhibitory motif (ITIM)-mediated recruitment of Src homology region 2 domain-containing phosphatase(SHP)-1 and SHP-2. Engagement of this receptor with its ligand has an important regulatory role in the prevention of excessive immune responses against infections and the maintenance of peripheral tolerance against self-antigens (reviewed in [59,60]). However the biology of PD-1:PD-L1/L2 is perhaps more complex than a uni-directional co-inhibitory pathway (summarized in Figure 1). Indeed, signaling through PD‑L1 in tumor cells enhances resistance to apoptosis [61]. In addition, hyper activated immunity in response to influenza [62] and *listeria monocytogenes* [63] in the absence of PD-L1 mediated signals has been reported, with authors suggesting PD-L1 mediates conditioning of APCs and/or the presence of a stimulatory PD-L1-induced signal in these models. Interestingly, PD-1 deficient mice are extremely sensitive to *Mycobacterium tuberculosis* infection exhibiting heightened pro-inflammatory responses yet, paradoxically, reduced T and B cell responses and uncontrolled bacterial proliferation [64]. Thus, the role that PD-1 plays during infections *in vivo* can be complex. 

In the context of acute viral infections, with the exception of influenza, PD-1/PD-L1 interactions inhibit antiviral T cell immunity [65]. Importantly, PD-1 signaling also drives T cell dysfunction and virus persistence in chronic LCMV infection [66]. Furthermore, blockade of PD-1:PD-L1 interactions improves the function of T cells reactive to human immunodeficiency virus (HIV) [67] and hepatitis B virus (HBV) [68]. Interestingly, PD-1 is up-regulated by monocytes during HIV infection, and ligation of this inhibitory receptor by PD-L1 induces IL-10 secretion and leads to CD4^+^ T cell dysfunction [69]. 

The PD-1:PD-L1/L2 pathway is a potentially critical regulator of anti-herpesvirus immunity. EBV‑specific CD8^+^ T cells up-regulate PD-1 upon activation [67], and a negative correlation between intensity of PD-1 expression and absolute numbers of circulating EBV-specific CD8^+^ T cells in the transition from acute infectious mononucleosis to convalescence has been reported [70]. Furthermore, PD-1 mediated exhaustion of EBV-specific (but not HCMV-specific) CD8^+^ T cells is detected in Systemic Lupus Erythematosus [71]. PD-1 ligation also suppresses HCMV-specific CD8^+^ T cell function [72] and has been implicated in driving attrition of CMV-specific CD8^+^ T cells in acute hepatitis B virus‑infected individuals [73]. 

These data suggest that PD-1 ligation on herpesvirus-specific T cells may antagonize control of infection *in vivo*; a hypothesis tested in murine models of herpesvirus infections. Although MCMV‑specific CD8^+^ T cells express PD-1 in a major site of persistence, the salivary glands, PD-L1 blockade failed to accelerate virus clearance [74]. Importantly, salivary gland-infiltrating antigen-specific CD8^+^ T cells are not exposed to cognate antigen due to a combination of efficient viral interference with MHC class I antigen processing in infected glandular epithelial cells and the inability of salivary gland APCs to cross-present antigen to CD8^+^ T cells [75]. Thus, any possible benefit of blockade of the PD-L1 pathway in this setting will be masked by impaired stimulation of virus-specific CD8^+^ T cells. Importantly, in a model of chronic murine γ-herpesvirus-68 (MHV-68) infection in MHC class II‑deficient mice in which PD-1 expression by virus-specific CD8^+^ T cells is observed, blockade of the PD-1:PD-L1 pathway reduces virus reactivation [76], underlining the potential importance of this inhibitory pathway in the suppression of protective anti-herpesvirus immunity. 

The potential suppressive activity of this pathway represents an obvious target for herpesviruses to exploit. Although no herpesvirus proteins with homology to PD-1 ligands have been reported, MCMV infection of dendritic cells induces surface expression of both PD-L1 and PD-L2 [77,78]. In stark contrast to virus-induced down-regulation of stimulatory ligands (CD80, CD86, CD40), adhesion molecules (CD11b and CD11c) and MHC Class I and II, prolonged PD-L1 up-regulation is observed in infected cells [78]. Strikingly, poor activation of T cells by MCMV-infected DCs is reversed by PD-L1 blockade *in vitro* and *in vivo*, suggesting MCMV actively manipulates PD-L1 to inhibit T cell activation [78]. Interestingly, tumor cells in EBV-associated cancers such as classical Hodgkin’s Lymphoma (cHL) and post-transplant lymphoproliferative disorders also express significant levels of PD-L1 [79] suggesting possible active manipulation of this pathway by EBV. 

Therefore, current data suggest it is beneficial for herpesviruses to exploit the PD-1:PD-L1/L2 inhibitory pathway. It is unclear whether these viruses specifically target the inhibition of antiviral T cell responses, or whether PD-1 mediated inhibition of other immune effector mechanisms may be exploited by these viruses. Furthermore, the potential virus exploitation of anti-apoptotic signaling events through PD-L1 in infected cells requires further investigation. 

## 4. B and T Lymphocyte Attenuator (BTLA)

Like PD-1, BTLA (or CD272) is a CD28 family member. As with PD-1, BTLA contains an ITIM and an immunoreceptor tyrosine-based switch motif (ITSM) in the membrane proximal and distal regions of the cytoplasmic domain, respectively. Whereas the negative signal induced by PD-1 ligation is predominantly transmitted through ITSM recruitment of SHP-1 and SHP-2 [80], both ITIM and ITSM motifs appear critical for BTLA-mediated suppression of T cell activation [81]. In addition, BTLA contains a Grb-2 recognition consensus site that may contribute to negative signaling [81]. 

BTLA expression was first reported on Th1 cells but is known to be expressed on a broad array of hematopoietic cells [82]. Studies of BTLA-deficient mice have highlighted an important regulatory role for BTLA in limiting mucosal inflammation and autoimmunity (reviewed in [82,83]) and BTLA limits immune responsiveness to bacterial and parasitic infections [84,85], suggesting a classical inhibitory function for this CD28-family member. However, the biology of BTLA is complicated by positive signaling delivered through the cellular ligand of BTLA; the tumor necrosis factor receptor superfamily member (TNFRSF) herpesvirus entry mediator (HVEM, or TNFRSF14). BTLA:HVEM interactions induce bidirectional signaling resulting in HVEM-mediated NF-kappaB activation (Figure 1). This signal promotes survival of HVEM-expressing cells, thus demonstrating that BTLA can also promote T cell responses [86]. 

HCMV, however, has evolved to exploit the inhibitory properties of BTLA. UL144 is a virus‑encoded truncated TNFR member consisting of two cysteine-rich domains (CRDs) homologous to CRD1 and CRD2 of HVEM [87]. Despite sequence hypervariation in the ectodomain of UL144 variants expressed by clinical HCMV isolates [88], UL144 proteins from all groups bind BTLA and, critically, UL144 suppresses proliferation of polyclonally stimulated CD4^+^ T cells [87]. Furthermore, BTLA is highly expressed by HCMV-specific CD8^+^ T cells following activation and BTLA blockade enhances HCMV-specific CD8^+^ T cell proliferation *in vitro* [89]. Therefore, through the acquisition and retention of UL144, HCMV appears to induce inhibitory signals induced downstream of BTLA to suppress antiviral T cell immunity (Figure 1). Given the broad expression pattern of BTLA on hematopoietic cells, it is conceivable that HCMV has evolved to target this inhibitory pathway to antagonize multiple immune effector mechanisms. 

## 5. CD200:CD200 Receptor Pathway

CD200 is a member of the immunoglobulin superfamily (IgSF) expressed on membranes by a heterogeneous group of cells, including B cells, activated T cells, endothelial cells, epithelial cells, follicular dendritic cells and neurons [90]. The expression of CD200 in humans [91], mice [92] and rats [93] is highly conserved. CD200 contains two extracellular IgSF domains, a hydrophobic trans-membrane sequence, and a short cytoplasmic domain [94] that does not contain known signaling motifs or docking sites for adaptor proteins [91]. Thus, CD200 is thought to deliver a unidirectional signal to its cellular receptor, CD200R.

Expression of CD200R was originally detected on myeloid cells, including macrophages, granulocytes and dendritic cells [95], but has subsequently been detected on NK cells and T cells [96,97]. CD200R contains two IgSF domains and a cytoplasmic region containing two tyrosine based motifs that can be phosphorylated [98]. In myeloid cells the inhibitory signal induced by CD200R is facilitated by recruitment of DOK2 and RasGAP [99]. Upon interaction with CD200, CD200R delivers an immunosuppressive signal that antagonizes type 1 cytokine production by DCs [100], induces Treg development [101] and inhibits macrophage function [92,98]. Moreover, CD200:CD200R limits myeloid cell homeostasis in the periphery [92], lung [102] and, to a lesser extent, the intestinal mucosa [103]. CD200R is also a critical negative regulator of inflammation. CD200-deficient mice demonstrate rapid onset of experimental autoimmune encephalomyelitis [98] and increased susceptibility to acute inflammation induced by bacterial (*Neisseria meningitidis*) [104] and viral (influenza) [102] infections. Importantly, therapeutic ligation of CD200 during ocular HSV infection also reduced inflammatory lesions [105], demonstrating the anti-inflammatory nature of this pathway in an acute herpesvirus infection.

### Viral CD200 Homologues (vCD200s)

The critical role for the CD200:CD200R pathway in the control of homeostasis and inflammation suggests an important regulatory function for this ligand-receptor pair in mammals. Importantly however, virus-encoded homologues of CD200 (vCD200s) have been identified in the genomes of several evolutionary diverse viruses, including herpesviruses, poxviruses, and adenoviruses, suggesting that exploitation of the mammalian CD200R pathway increases evolutionary fitness of viruses [106].

**Figure 2:**
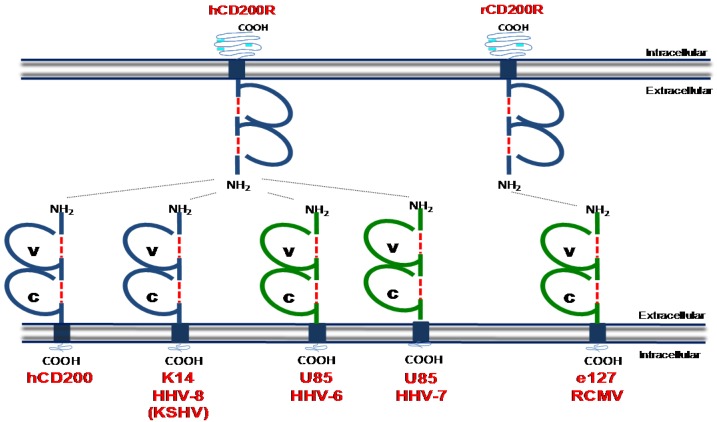
Determined structures of cellular CD200 and CD200 homologues encoded by human and rat herpesviruses. ‘V’ denotes a conserved variable-like Ig domain and ‘C’ denotes a conserved constant-like Ig domain. These domains are formed by disulfide binds which are denoted by a red broken line. Light blue portions of the intracellular domain of CD200R denotes tyrosine residues which can be phosphorylated during intracellular signalling. Dashed lines between receptor/ligands represent known interaction as detected in biochemical binding assays. Blue = structure known, Green = predicted structure based on homology to cellular CD200. Structural data was obtained from [112].

Members of the β-herpesviridae (HHV-6a, HHV-6b and HHV7) and γ-herpesviridae (HHV‑8/KSHV and Rhesus rhadinovirus) encode vCD200s (Figure 2). Sequence homology of vCD200s with mammalian proteins suggests that viruses acquired these genes from host cells [106]. KSHV encodes the best-characterized vCD200, K14, which binds to hCD200R with almost identical kinetics as mammalian (human) CD200 despite exhibiting only 40% sequence identify to its mammalian counterpart [106]. K14 is expressed during the lytic phase of HHV-8 replication [106]. *In vitro* experiments utilizing K14-transfected cells or K14 fusion proteins have indicated that K14 suppresses the activation of neutrophils [107], basophils and NK cells [108], T cells [109] and macrophages [106]. Paradoxically however, CD200 has been reported to activate myeloid cells under certain experimental conditions [110,111]. Whether differences in these results represent the different experimental approaches taken (fusion proteins *versus* cell transfection) or whether the outcome of K14 signaling is determined by the “infected” K14-transfected cell, is unclear. 

CD200 homologues encoded by HHV-6 [113] and HHV-7 [114] also bind hCD200R [108], although the functional consequences of this interaction in hematopoietic cells is unclear. The Rhesus rhadinovirus (RRV) R15 ORF exhibits 30% homology to human CD200 and is detected in the cytoplasm and on the cell surface during infection. When expressed as a fusion protein, RRV R15 inhibits pro-inflammatory cytokine expression by myeloid cells consistent with an immune suppressive function for vCD200s. Finally, the English isolate of rat cytomegalovirus (RCMV-E) encodes a CD200 homologue (e127) [115] which binds rat CD200R [116]. Intriguingly, absence of e127 expression by RCMV-E does not influence the induction of pro-inflammatory cytokine expression *in vitro*. However, the authors reported a correlation between the absence of e127 and heightened myeloid cell activity *in vivo* [116], suggesting cytomegalovirus may exploit this pathway to suppress myeloid cell activation. In support of this hypothesis, CD200R deficient mice exhibit improved control of MCMV infection and increased immune cell responsiveness (including myeloid cells) in infected organs [117]. Interestingly however, sequencing of multiple MCMV genomes including three low‑passage strains isolated from wild mice [118] and the commonly used laboratory Smith strain [119] has yet to identify a CD200 homologue in MCMV. 

Why do closely related β-herpesviruses vary in their exploitation of this pathway? Interestingly, the murine CD200R (mCD200R) family contains four additional genes and a pseudogene, although the repertoire of these genes varies in different mouse strains [120]. Proteins encoded by these genes, termed CD200R-like proteins a–e (CD200Rla–e) have predicted cytoplasmic binding sites for the signaling molecule DAP12, suggesting activating properties [97]. Although mCD200 does not interact with activating CD200R family members [121], it is conceivable that evolution of activating mCD200R family members may have pressurized MCMV to avoid acquisition of a CD200-like gene due to potential cross-reactivity with these activating receptors. Although a similar activating CD200R gene is encoded within the human genome, it is not expressed on the cell surface [97]. It is unknown whether this protein is in the process of being selected into the human population or is being eradicated. Thus the possible influence of this human gene on the past acquisition (or lack of) of vCD200 by different herpesviruses is currently unclear. Irrespective of the possible presence of activating CD200R-like molecules in some mammals, taken with available literature on pox viruses [122], the majority of data suggests that multiple herpesviruses have acquired vCD200 molecules to inhibit virus-induced immune responses.

## 6. Mammalian and Viral Immune Inhibitory Molecules: Therapeutic Targets or Necessary Evils?

Immune inhibitory receptors represent important targets for herpesviruses to dampen antiviral immunity. Subsequently, these pathways or the virus-encoded proteins that exploit them represent possible targets of therapeutic strategies aiming to promote antiviral immunity. 

The therapeutic success of specifically targeting viral homologues of immune-modulatory proteins will be partially determined by the sequence homology to their mammalian counterparts. Relatively low amino acid sequence similarity between HCMV UL111A and mammalian IL-10 [19] offers the potential of targeting UL111A in vaccine settings, as demonstrated with rhCMV UL111A [123,124]. Such an approach however is considerably more problematic for EBV due to the high sequence homology between BCRF-1 and mammalian IL-10 [20]. Moreover hypervariation in some viral proteins, for example in UL144 [88], represents further challenges for vaccination strategies targeting these molecules. 

Importantly, *in vivo* data (predominantly from murine models of virus infections) highlight the important role of inhibitory pathways in limiting infection-induced pathology, particularly in acute infections. Therefore, herpesvirus persistence and dissemination that occurs as a consequence of signaling through these receptors is, from an evolutionary perspective, perhaps an acceptable price for mammals to pay to ensure effective control of virus-elicited immune-mediated damage. Thus, therapeutic treatment with antagonists of mammalian immune-inhibitory proteins during acute infections may have harmful consequences. Paradoxically, in certain conditions, ligation of inhibitory receptors may improve virus-induced pathology [46,105]. Importantly, to facilitate establishment of chronic infection and dissemination from their host, herpesviruses are also dependent upon survival and fitness of the mammal they infect. Therefore, an important function of viral proteins that target these pathways may be to contribute to the limitation of infection-induced pathology. *In vivo* infection models that utilize herpesviruses encoding such immune evasion proteins [51,116] will be particularly powerful tools to assess this possibility. 

Much of the literature summarized herein implies that herpesviruses target immune inhibitory pathways to antagonize T cell responses. HCMV, however, induces the generation of a remarkably large virus-specific T cell response over time [125] suggesting that the primary function of these immune evasion strategies is not to antagonize memory T cell development. Importantly however, experiments using the MCMV model have demonstrated that memory T cell inflation is greatly increased in the absence of IL-10 [49], implying that inhibitory immune pathways could be exploited to further enhance the functional capabilities and/or *in vivo* survival of herpesvirus-specific memory T cells used, for example, in T cell-based therapies to treat herpesvirus-infected individuals. 

It is likely that herpesviruses exploit immune inhibitory pathways to antagonize T cell responses primarily during acute infection, and during persistence in mucosal tissues, thus promoting virus survival and dissemination. Although targeting these pathways during acute infection may have harmful consequences (as discussed above), manipulation of these molecules to enhance virus-specific immunity induced by prophylactic vaccination strategies (including, possibly, attenuated herpesviruses) may greatly improve anti-herpesvirus immunity. Importantly, the observation that re‑infection of HCMV-seropositive women with different strains of HCMV can lead to intrauterine transmission of the virus [126] suggests that, in the case of this herpesvirus, boosting existing HCMV‑specific T cell responses, particularly in mucosal surfaces, may be crucial to reduce horizontal transmission and resulting congenital infections. Thus, understanding the role that immune inhibitory proteins play in regulating anti-herpesvirus immunity in mucosal (and non-mucosal) tissues of persistent/chronically infected hosts may inform strategies aiming to enhance heterosubtypic immunity afforded by existing herpesvirus-specific T cell responses. *In vivo* experiments will be crucial for the assessment of the efficacy and safety of targeting immune-inhibitory pathways to enhance herpesvirus-specific protective immunity in all of these settings. 

## Supplementary Files

Supplementary File 1: PDF-Document (PDF, 124 KB)
