# Supplementary figures and images for: Herpesvirus Exploitation of Host Immune Inhibitory Pathways
Source: Viruses. 2012 Aug 3;4(8):1182–201. doi: 10.3390/v4081182 (PMC3446756; doi:10.3390/v4081182)

Figure 1

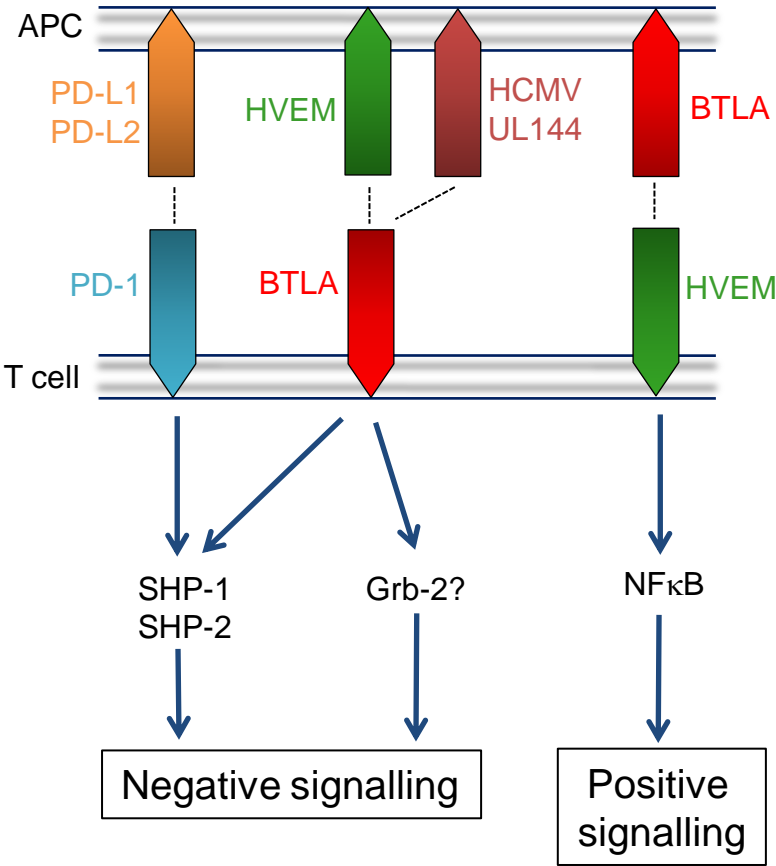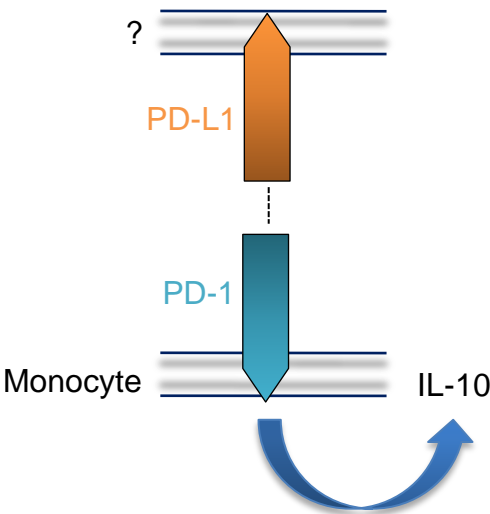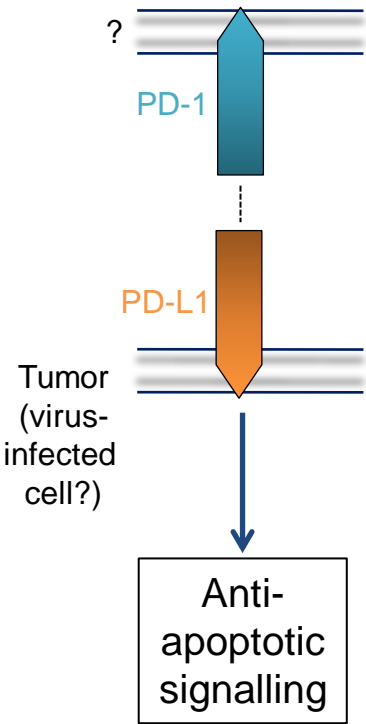

Figure 2

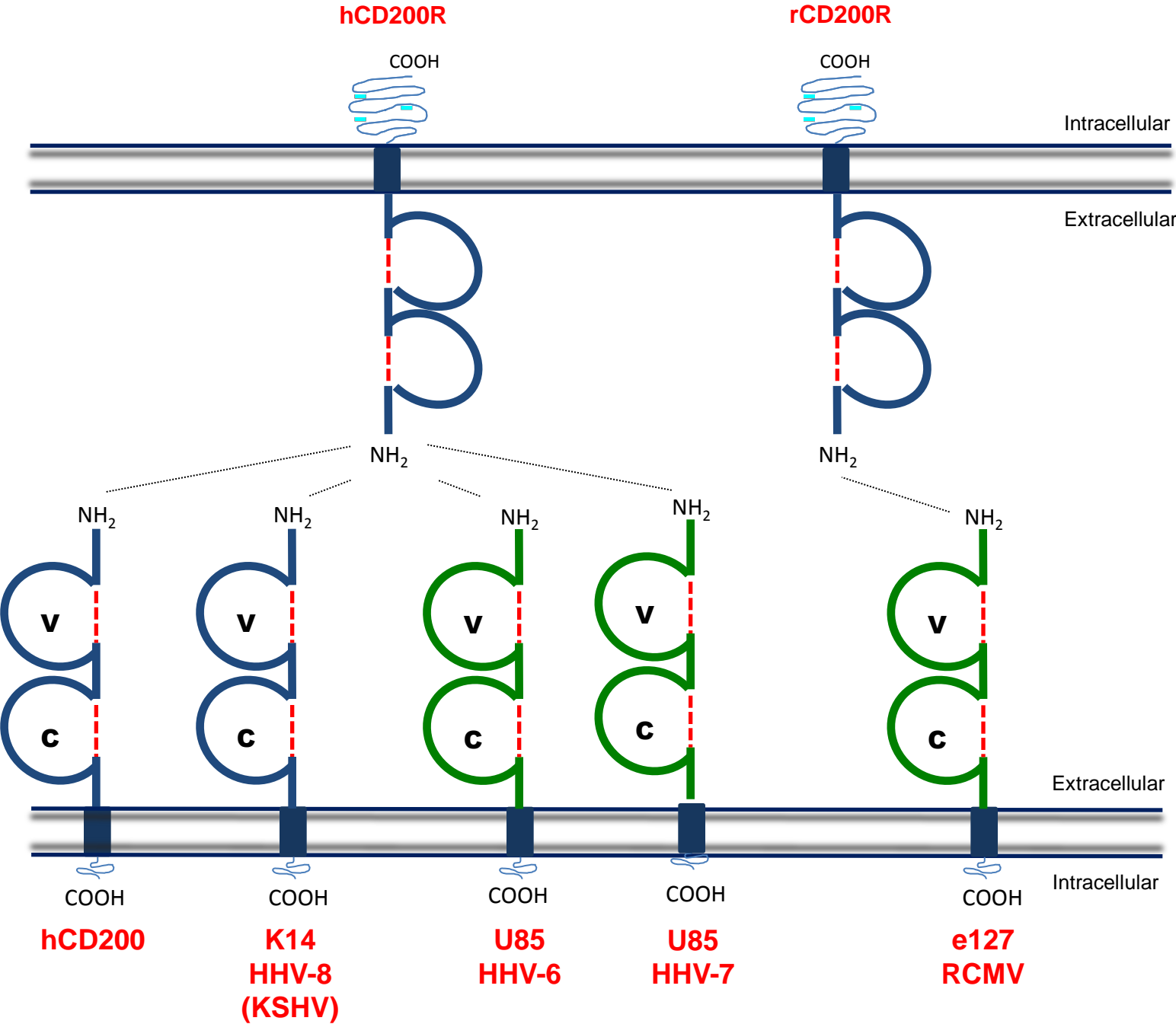

Supplement: Supplementary File 1: — PDF-Document (PDF, 124 KB) [file viruses-04-01182-s001.pdf]
